# (*E*)-1-(4,4′′-Difluoro-5′-meth­oxy-1,1′:3′,1′′-terphenyl-4′-yl)-3-(6-meth­oxy­naphthalen-2-yl)prop-2-en-1-one

**DOI:** 10.1107/S1600536811047660

**Published:** 2011-11-16

**Authors:** Hoong-Kun Fun, Madhukar Hemamalini, S. Samshuddin, B. Narayana, B. K. Sarojini

**Affiliations:** a X-ray Crystallography Unit, School of Physics, Universiti Sains Malaysia, 11800 USM, Penang, Malaysia; b Department of Studies in Chemistry, Mangalore University, Mangalagangotri 574 199, India; c Department of Chemistry, P. A. College of Engineering, Nadupadavu, Mangalore 574 153, India

## Abstract

In the title compound, C_33_H_24_F_2_O_3_, the central benzene ring makes dihedral angles of 44.71 (10), 47.80 (10) and 63.68 (9)° with the two fluoro-substituted benzene rings and the naphthalene ring system, respectively. In the crystal, mol­ecules are connected *via* inter­molecular C—H⋯F and C—H⋯O hydrogen bonds. Furthermore, the crystal structure is stabilized by weak C—H⋯π and π–π inter­actions [centroid–centroid distance = 3.6816 (13) Å].

## Related literature

For applications of chalcones, see: Dhar (1981 ▶); Dimmock *et al.* (1999 ▶); Satyanarayana *et al.* (2004 ▶); Sarojini *et al.* (2006 ▶); Liu (2006 ▶); Astruc (2002 ▶). For related structures, see: Samshuddin, Narayana *et al.* (2011 ▶); Samshuddin, Butcher *et al.* (2011 ▶); Fun *et al.* (2010**a* ▶,b* ▶); Jasinski *et al.* (2010**a* ▶,b* ▶); Baktır *et al.* (2011**a* ▶,b* ▶).
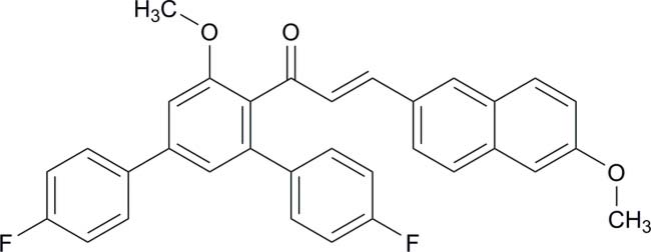


## Experimental

### 

#### Crystal data

C_33_H_24_F_2_O_3_
*M* *_r_* = 506.52
Monoclinic, 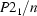

*a* = 6.9524 (5) Å
*b* = 33.024 (2) Å
*c* = 11.6030 (9) Å
β = 107.267 (1)°
*V* = 2544.0 (3) Å^3^
*Z* = 4
Mo *K*α radiation
μ = 0.09 mm^−1^
*T* = 296 K
0.36 × 0.16 × 0.08 mm

#### Data collection

Bruker APEXII DUO CCD area-detector diffractometer
Absorption correction: multi-scan (*SADABS*; Bruker, 2009 ▶) *T*_min_ = 0.967, *T*_max_ = 0.993
53824 measured reflections
7441 independent reflections
4312 reflections with *I* > 2σ(*I*)
*R*_int_ = 0.056

#### Refinement

*R*[*F* ^2^ > 2σ(*F* ^2^)] = 0.065
*wR*(*F* ^2^) = 0.180
*S* = 1.02
7441 reflections
345 parameters
H-atom parameters constrained
Δρ_max_ = 0.28 e Å^−3^
Δρ_min_ = −0.18 e Å^−3^

### 

Data collection: *APEX2* (Bruker, 2009 ▶); cell refinement: *SAINT* (Bruker, 2009 ▶); data reduction: *SAINT*; program(s) used to solve structure: *SHELXTL* (Sheldrick, 2008 ▶); program(s) used to refine structure: *SHELXTL*; molecular graphics: *SHELXTL*; software used to prepare material for publication: *SHELXTL* and *PLATON* (Spek, 2009 ▶).

## Supplementary Material

Crystal structure: contains datablock(s) global, I. DOI: 10.1107/S1600536811047660/is5005sup1.cif

Structure factors: contains datablock(s) I. DOI: 10.1107/S1600536811047660/is5005Isup2.hkl

Supplementary material file. DOI: 10.1107/S1600536811047660/is5005Isup3.cml

Additional supplementary materials:  crystallographic information; 3D view; checkCIF report

## Figures and Tables

**Table 1:** Hydrogen-bond geometry (Å, °) *Cg*1, *Cg*3 and *Cg*4 are the centroids of the C1–C3/C8–C10, C14–C19 and C20–C25 rings, respectively.

*D*—H⋯*A*	*D*—H	H⋯*A*	*D*⋯*A*	*D*—H⋯*A*
C28—H28*A*⋯O2^i^	0.93	2.53	3.363 (3)	148
C32—H32*C*⋯F1^ii^	0.96	2.40	3.275 (4)	152
C33—H33*A*⋯F2^iii^	0.96	2.48	3.404 (3)	162
C32—H32*A*⋯*Cg*1^iv^	0.96	2.82	3.767 (4)	168
C24—H24*A*⋯*Cg*3^v^	0.93	2.83	3.461 (3)	126
C33—H33*B*⋯*Cg*3^vi^	0.96	2.91	3.556 (3)	126
C7—H7*A*⋯*Cg*4^iii^	0.93	2.85	3.548 (3)	133

Symmetry codes: (i) 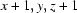; (ii) 
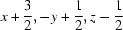
; (iii) 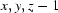; (iv) 
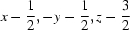
; (v) 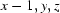; (vi) 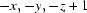.

**Fig. 1.**
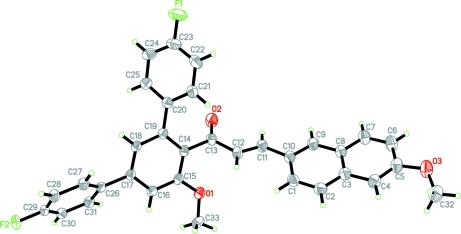
An ORTEP view of the title compound, showing 30% probability displacement ellipsoids.

**Fig. 2.**
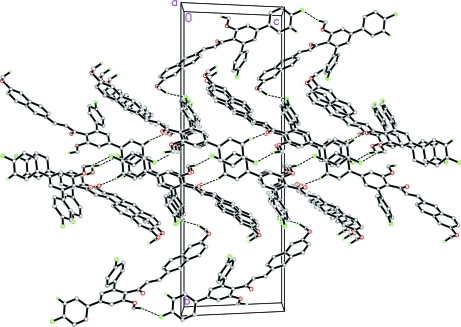
The crystal packing diagram of the title compound. H atoms are not involving the hydrogen bond interactions are omitted for clarity.

## supplementary crystallographic information

### Comment

Chalcones are highly reactive substances of varied nature. They have been reported to possess many interesting pharmacological properties (Dhar, 1981) including anti-inflammatory, antimicrobial, antifungal, antioxidant, cytotoxic, antitumor and anticancer activities (Dimmock *et al.*, 1999; Satyanarayana *et al.*, 2004). Chalcones also find application as organic nonlinear optical materials (NLO) for their SHG conversion efficiency (Sarojini *et al.*, 2006). The basic skeleton of chalcones which possess α,β-unsaturated carbonyl group is a useful synthone for the synthesis of various biodynamic cyclic derivatives such as  pyrazoline, benzodiazepine, 2,4,6-triarylpyridine, isoxazoline and cyclohexenone derivatives.  Polysubstituted aromatics are key structures of great efficacy in synthetic, medicinal and natural product chemistry. In recent years, it has been reported that some terphenyls exhibit considerable biological activities, *e.g.*, potent anticoagulant, immunosuppressants, anti-thrombotic, neuroprotective, specific 5-lipoxygenase inhibitory and cytotoxic activities (Liu, 2006). Due to their promising biological activities and important properties, terphenyls have generated increasing research interests. As such, the synthesis of polysubstituted aromatics has been a fascinating area in organic chemistry (Astruc, 2002).

In view of the pharmacological importance of terphenyls and chalcones, and in continuation of our work on synthesis of various derivatives of 4,4'-difluorochalcone (Samshuddin, Narayana *et al.*, 2011; Samshuddin, Butcher *et al.*, 2011; Fun *et al.*, 2010**a*,b*; Jasinski *et al.*, 2010**a*,b*; Baktır *et al.*, 2011**a*,b*), the title compound (I) is prepared and its crystal structure is reported. The precursor of the title compound was prepared from 4,4'-difluorochalcone using several steps.

The asymmetric unit of the title compound as shown in Fig. 1. The naphthalene (C1–C10) ring system is approximately planar with a maximum deviation of 0.055 (2) Å for atom C10.  The central benzene (C14–C19) ring makes dihedral angles of 44.71 (10), 47.80 (10) and 63.68 (9)° with the attached two fluoro-substituted benzene (C20–C25 and C26–C31) rings and the naphthalene (C1–C10) ring system, respectively.

In the crystal structure, (Fig. 2), the molecules are connected *via* intermolecular C—H···F and C—H···O hydrogen bonds. Furthermore, the crystal structure is stabilized by a weak π–π interaction between the benzene (C26–C31) rings [*Cg*···*Cg*(-*x*, -*y*, 2 - *z*) = 3.6816 (13) Å] and C—H···π (Table 1) interactions, involving the centroids of the C1–C3/C8–C10 (*Cg*1), C14–C19 (*Cg*3) and C20–C25 (*Cg*4) rings.

### Experimental

To  a mixture of 1-(4,4''-difluoro-5'-methoxy-1,1':3',1''-terphenyl-4'-yl) ethanone (0.338 g, 0.001 mol) and 6-methoxy-2-naphthaldehyde (0.188 g, 0.001 mol) in 30 ml ethanol, 0.5 ml of  10% sodium hydroxide solution  was added and stirred at 5–10 °C for 3 hours. The precipitate formed was collected by filtration and purified by recrystallization from ethanol. Single crystals were grown from DMF by slow evaporation method and the yield of the compound was 84% (m.p. 479K).

### Refinement

All hydrogen atoms were positioned geometrically (C—H = 0.93 or 0.96 Å) and were refined using a riding model, with *U*_iso_(H) = 1.2*U*_eq_(C) or 1.5*U*_eq_(methyl C). A rotating group model was applied to the methyl groups.

### Crystal data

**Table:**

C_33_H_24_F_2_O_3_	*F*(000) = 1056
*M**_r_* = 506.52	*D*_x_ = 1.322 Mg m^−^^3^
Monoclinic, *P*2_1_/*n*	Mo *K*α radiation, λ = 0.71073 Å
Hall symbol: -P 2yn	Cell parameters from 7590 reflections
*a* = 6.9524 (5) Å	θ = 2.5–26.5°
*b* = 33.024 (2) Å	µ = 0.09 mm^−^^1^
*c* = 11.6030 (9) Å	*T* = 296 K
β = 107.267 (1)°	Plate, yellow
*V* = 2544.0 (3) Å^3^	0.36 × 0.16 × 0.08 mm
*Z* = 4	

### Data collection

**Table:**

Bruker APEXII DUO CCD area-detector diffractometer	7441 independent reflections
Radiation source: fine-focus sealed tube	4312 reflections with *I* > 2σ(*I*)
graphite	*R*_int_ = 0.056
φ and ω scans	θ_max_ = 30.1°, θ_min_ = 1.9°
Absorption correction: multi-scan (*SADABS*; Bruker, 2009)	*h* = −9→9
*T*_min_ = 0.967, *T*_max_ = 0.993	*k* = −46→46
53824 measured reflections	*l* = −16→16

### Refinement

**Table:**

Refinement on *F*^2^	Primary atom site location: structure-invariant direct methods
Least-squares matrix: full	Secondary atom site location: difference Fourier map
*R*[*F*^2^ > 2σ(*F*^2^)] = 0.065	Hydrogen site location: inferred from neighbouring sites
*wR*(*F*^2^) = 0.180	H-atom parameters constrained
*S* = 1.02	*w* = 1/[σ^2^(*F*_o_^2^) + (0.0622*P*)^2^ + 1.2079*P*] where *P* = (*F*_o_^2^ + 2*F*_c_^2^)/3
7441 reflections	(Δ/σ)_max_ = 0.001
345 parameters	Δρ_max_ = 0.28 e Å^−^^3^
0 restraints	Δρ_min_ = −0.18 e Å^−^^3^

### Special details

**Table:**

Geometry. All s.u.'s (except the s.u. in the dihedral angle between two l.s. planes) are estimated using the full covariance matrix. The cell s.u.'s are taken into account individually in the estimation of s.u.'s in distances, angles and torsion angles; correlations between s.u.'s in cell parameters are only used when they are defined by crystal symmetry. An approximate (isotropic) treatment of cell s.u.'s is used for estimating s.u.'s involving l.s. planes.
Refinement. Refinement of F^2^ against ALL reflections. The weighted R-factor wR and goodness of fit S are based on F^2^, conventional R-factors R are based on F, with F set to zero for negative F^2^. The threshold expression of F^2^ > 2σ(F^2^) is used only for calculating R-factors(gt) etc. and is not relevant to the choice of reflections for refinement. R-factors based on F^2^ are statistically about twice as large as those based on F, and R- factors based on ALL data will be even larger.

### Fractional atomic coordinates and isotropic or equivalent isotropic displacement parameters (Å^2^)

**Table:**

	*x*	*y*	*z*	*U*_iso_*/*U*_eq_	
F1	−0.8506 (3)	0.20150 (6)	0.53664 (18)	0.1044 (6)	
F2	0.3881 (2)	−0.01095 (5)	1.20226 (11)	0.0746 (4)	
O1	0.0952 (3)	0.04503 (5)	0.43318 (13)	0.0590 (4)	
O2	−0.4053 (3)	0.08801 (6)	0.33233 (14)	0.0719 (5)	
O3	0.1800 (3)	0.25775 (6)	−0.26009 (18)	0.0868 (6)	
C1	0.1517 (4)	0.15151 (8)	0.1660 (2)	0.0595 (6)	
H1A	0.2255	0.1415	0.2409	0.071*	
C2	0.2463 (4)	0.17487 (8)	0.1012 (2)	0.0616 (6)	
H2A	0.3830	0.1805	0.1328	0.074*	
C3	0.1381 (4)	0.19040 (7)	−0.0126 (2)	0.0555 (5)	
C4	0.2299 (4)	0.21664 (7)	−0.0797 (2)	0.0623 (6)	
H4A	0.3652	0.2236	−0.0495	0.075*	
C5	0.1152 (4)	0.23119 (8)	−0.1890 (2)	0.0649 (7)	
C6	−0.0870 (5)	0.22052 (8)	−0.2378 (2)	0.0725 (7)	
H6A	−0.1610	0.2305	−0.3127	0.087*	
C7	−0.1757 (4)	0.19549 (8)	−0.1758 (2)	0.0660 (7)	
H7A	−0.3102	0.1883	−0.2090	0.079*	
C8	−0.0662 (4)	0.18022 (7)	−0.06174 (19)	0.0515 (5)	
C9	−0.1600 (4)	0.15587 (7)	0.00749 (19)	0.0555 (5)	
H9A	−0.2949	0.1489	−0.0249	0.067*	
C10	−0.0563 (4)	0.14246 (7)	0.12064 (18)	0.0521 (5)	
C11	−0.1675 (4)	0.12159 (7)	0.19354 (19)	0.0538 (5)	
H11A	−0.2986	0.1135	0.1536	0.065*	
C12	−0.0997 (4)	0.11319 (7)	0.30973 (19)	0.0537 (5)	
H12A	0.0334	0.1194	0.3510	0.064*	
C13	−0.2262 (3)	0.09428 (7)	0.37715 (18)	0.0488 (5)	
C14	−0.1234 (3)	0.08339 (6)	0.50667 (16)	0.0416 (4)	
C15	0.0403 (3)	0.05689 (6)	0.53151 (16)	0.0433 (4)	
C16	0.1302 (3)	0.04262 (6)	0.64713 (17)	0.0436 (4)	
H16A	0.2366	0.0244	0.6615	0.052*	
C17	0.0615 (3)	0.05552 (6)	0.74161 (16)	0.0403 (4)	
C18	−0.0957 (3)	0.08305 (6)	0.71871 (17)	0.0424 (4)	
H18A	−0.1376	0.0926	0.7827	0.051*	
C19	−0.1932 (3)	0.09687 (6)	0.60211 (17)	0.0405 (4)	
C20	−0.3657 (3)	0.12534 (6)	0.58344 (17)	0.0441 (4)	
C21	−0.3847 (4)	0.15999 (7)	0.5133 (2)	0.0554 (6)	
H21A	−0.2871	0.1660	0.4758	0.066*	
C22	−0.5472 (4)	0.18581 (8)	0.4985 (2)	0.0678 (7)	
H22A	−0.5595	0.2090	0.4514	0.081*	
C23	−0.6886 (4)	0.17661 (9)	0.5542 (2)	0.0660 (7)	
C24	−0.6756 (3)	0.14343 (8)	0.6256 (2)	0.0608 (6)	
H24A	−0.7733	0.1382	0.6637	0.073*	
C25	−0.5132 (3)	0.11777 (7)	0.63984 (19)	0.0498 (5)	
H25A	−0.5020	0.0949	0.6882	0.060*	
C26	0.1510 (3)	0.03850 (6)	0.86460 (16)	0.0418 (4)	
C27	0.2048 (3)	0.06306 (7)	0.96664 (18)	0.0496 (5)	
H27A	0.1866	0.0909	0.9582	0.060*	
C28	0.2850 (3)	0.04658 (8)	1.08059 (18)	0.0548 (6)	
H28A	0.3216	0.0630	1.1487	0.066*	
C29	0.3089 (3)	0.00566 (8)	1.09028 (18)	0.0520 (5)	
C30	0.2575 (3)	−0.01981 (7)	0.9932 (2)	0.0526 (5)	
H30A	0.2748	−0.0477	1.0030	0.063*	
C31	0.1792 (3)	−0.00303 (7)	0.88003 (18)	0.0479 (5)	
H31A	0.1447	−0.0198	0.8127	0.058*	
C32	0.3839 (5)	0.26621 (11)	−0.2300 (3)	0.0988 (11)	
H32A	0.4117	0.2808	−0.2948	0.148*	
H32B	0.4587	0.2413	−0.2164	0.148*	
H32C	0.4226	0.2823	−0.1580	0.148*	
C33	0.2823 (4)	0.02460 (8)	0.4521 (2)	0.0634 (6)	
H33A	0.3081	0.0207	0.3761	0.095*	
H33B	0.2765	−0.0012	0.4890	0.095*	
H33C	0.3883	0.0406	0.5042	0.095*	

### Atomic displacement parameters (Å^2^)

**Table:**

	*U*^11^	*U*^22^	*U*^33^	*U*^12^	*U*^13^	*U*^23^
F1	0.0836 (12)	0.1138 (15)	0.1051 (14)	0.0507 (11)	0.0118 (10)	−0.0035 (11)
F2	0.0776 (10)	0.1075 (12)	0.0390 (7)	0.0182 (8)	0.0177 (6)	0.0204 (7)
O1	0.0732 (11)	0.0692 (10)	0.0393 (8)	0.0146 (8)	0.0242 (7)	−0.0037 (7)
O2	0.0602 (11)	0.1037 (14)	0.0438 (9)	−0.0166 (10)	0.0028 (8)	−0.0040 (9)
O3	0.0988 (16)	0.0832 (14)	0.0796 (13)	−0.0171 (12)	0.0282 (12)	0.0173 (11)
C1	0.0659 (15)	0.0654 (15)	0.0455 (12)	0.0092 (12)	0.0137 (11)	0.0029 (11)
C2	0.0569 (14)	0.0661 (15)	0.0601 (14)	0.0036 (12)	0.0150 (11)	−0.0029 (12)
C3	0.0653 (15)	0.0515 (12)	0.0513 (12)	0.0012 (11)	0.0196 (11)	−0.0072 (10)
C4	0.0676 (16)	0.0565 (14)	0.0654 (15)	−0.0064 (12)	0.0239 (12)	−0.0037 (12)
C5	0.0828 (18)	0.0569 (14)	0.0574 (14)	−0.0104 (13)	0.0246 (13)	−0.0021 (11)
C6	0.090 (2)	0.0680 (16)	0.0529 (14)	−0.0142 (15)	0.0108 (13)	0.0043 (12)
C7	0.0745 (17)	0.0665 (16)	0.0499 (13)	−0.0104 (13)	0.0073 (12)	−0.0013 (11)
C8	0.0647 (14)	0.0466 (11)	0.0427 (11)	−0.0016 (10)	0.0153 (10)	−0.0052 (9)
C9	0.0635 (14)	0.0567 (13)	0.0449 (11)	−0.0021 (11)	0.0139 (10)	−0.0034 (10)
C10	0.0687 (15)	0.0493 (12)	0.0399 (10)	0.0042 (11)	0.0185 (10)	−0.0038 (9)
C11	0.0653 (14)	0.0545 (13)	0.0414 (11)	0.0004 (11)	0.0154 (10)	−0.0033 (9)
C12	0.0654 (14)	0.0557 (13)	0.0393 (10)	−0.0033 (11)	0.0145 (10)	−0.0004 (9)
C13	0.0547 (13)	0.0538 (12)	0.0348 (10)	−0.0040 (10)	0.0086 (9)	−0.0044 (8)
C14	0.0445 (11)	0.0459 (11)	0.0334 (9)	−0.0073 (9)	0.0103 (8)	−0.0031 (8)
C15	0.0491 (12)	0.0479 (11)	0.0358 (9)	−0.0054 (9)	0.0172 (8)	−0.0057 (8)
C16	0.0415 (10)	0.0472 (11)	0.0435 (10)	−0.0009 (9)	0.0146 (8)	−0.0022 (8)
C17	0.0419 (10)	0.0450 (10)	0.0345 (9)	−0.0040 (8)	0.0121 (8)	−0.0020 (8)
C18	0.0456 (11)	0.0495 (11)	0.0343 (9)	−0.0035 (9)	0.0152 (8)	−0.0046 (8)
C19	0.0399 (10)	0.0448 (10)	0.0368 (9)	−0.0057 (8)	0.0114 (8)	−0.0031 (8)
C20	0.0434 (11)	0.0460 (11)	0.0390 (10)	−0.0036 (9)	0.0063 (8)	−0.0057 (8)
C21	0.0622 (14)	0.0557 (13)	0.0473 (12)	0.0010 (11)	0.0147 (10)	0.0038 (10)
C22	0.0798 (18)	0.0586 (15)	0.0572 (14)	0.0153 (13)	0.0081 (13)	0.0072 (11)
C23	0.0544 (14)	0.0714 (17)	0.0633 (15)	0.0186 (12)	0.0038 (12)	−0.0123 (13)
C24	0.0444 (13)	0.0715 (16)	0.0651 (15)	−0.0015 (11)	0.0141 (11)	−0.0153 (12)
C25	0.0463 (12)	0.0528 (12)	0.0500 (12)	−0.0052 (10)	0.0135 (9)	−0.0048 (9)
C26	0.0376 (10)	0.0529 (12)	0.0358 (9)	−0.0002 (9)	0.0124 (8)	−0.0006 (8)
C27	0.0533 (12)	0.0537 (12)	0.0412 (10)	−0.0007 (10)	0.0130 (9)	−0.0045 (9)
C28	0.0538 (13)	0.0751 (16)	0.0341 (10)	−0.0004 (11)	0.0109 (9)	−0.0076 (10)
C29	0.0442 (12)	0.0783 (16)	0.0361 (10)	0.0092 (11)	0.0158 (9)	0.0100 (10)
C30	0.0503 (13)	0.0584 (13)	0.0522 (12)	0.0073 (10)	0.0201 (10)	0.0082 (10)
C31	0.0486 (12)	0.0543 (12)	0.0425 (10)	0.0014 (10)	0.0160 (9)	−0.0025 (9)
C32	0.089 (2)	0.095 (2)	0.117 (3)	−0.0174 (19)	0.037 (2)	0.020 (2)
C33	0.0670 (16)	0.0695 (16)	0.0648 (14)	0.0023 (13)	0.0368 (13)	−0.0119 (12)

### Geometric parameters (Å, °)

**Table:**

F1—C23	1.360 (3)	C16—C17	1.386 (3)
F2—C29	1.367 (2)	C16—H16A	0.9300
O1—C15	1.363 (2)	C17—C18	1.385 (3)
O1—C33	1.423 (3)	C17—C26	1.487 (3)
O2—C13	1.216 (3)	C18—C19	1.398 (3)
O3—C5	1.369 (3)	C18—H18A	0.9300
O3—C32	1.384 (4)	C19—C20	1.489 (3)
C1—C2	1.373 (3)	C20—C21	1.387 (3)
C1—C10	1.416 (3)	C20—C25	1.393 (3)
C1—H1A	0.9300	C21—C22	1.385 (3)
C2—C3	1.408 (3)	C21—H21A	0.9300
C2—H2A	0.9300	C22—C23	1.362 (4)
C3—C8	1.406 (3)	C22—H22A	0.9300
C3—C4	1.435 (3)	C23—C24	1.360 (4)
C4—C5	1.369 (4)	C24—C25	1.382 (3)
C4—H4A	0.9300	C24—H24A	0.9300
C5—C6	1.396 (4)	C25—H25A	0.9300
C6—C7	1.358 (4)	C26—C31	1.389 (3)
C6—H6A	0.9300	C26—C27	1.392 (3)
C7—C8	1.409 (3)	C27—C28	1.385 (3)
C7—H7A	0.9300	C27—H27A	0.9300
C8—C9	1.424 (3)	C28—C29	1.362 (3)
C9—C10	1.370 (3)	C28—H28A	0.9300
C9—H9A	0.9300	C29—C30	1.366 (3)
C10—C11	1.476 (3)	C30—C31	1.379 (3)
C11—C12	1.319 (3)	C30—H30A	0.9300
C11—H11A	0.9300	C31—H31A	0.9300
C12—C13	1.478 (3)	C32—H32A	0.9600
C12—H12A	0.9300	C32—H32B	0.9600
C13—C14	1.504 (3)	C32—H32C	0.9600
C14—C15	1.396 (3)	C33—H33A	0.9600
C14—C19	1.407 (3)	C33—H33B	0.9600
C15—C16	1.384 (3)	C33—H33C	0.9600
			
C15—O1—C33	118.47 (17)	C17—C18—H18A	119.1
C5—O3—C32	118.2 (2)	C19—C18—H18A	119.1
C2—C1—C10	121.2 (2)	C18—C19—C14	118.51 (18)
C2—C1—H1A	119.4	C18—C19—C20	119.03 (17)
C10—C1—H1A	119.4	C14—C19—C20	122.46 (17)
C1—C2—C3	120.6 (2)	C21—C20—C25	117.9 (2)
C1—C2—H2A	119.7	C21—C20—C19	122.60 (19)
C3—C2—H2A	119.7	C25—C20—C19	119.46 (19)
C8—C3—C2	119.2 (2)	C22—C21—C20	120.9 (2)
C8—C3—C4	118.9 (2)	C22—C21—H21A	119.6
C2—C3—C4	121.9 (2)	C20—C21—H21A	119.6
C5—C4—C3	118.9 (2)	C23—C22—C21	118.8 (2)
C5—C4—H4A	120.5	C23—C22—H22A	120.6
C3—C4—H4A	120.5	C21—C22—H22A	120.6
O3—C5—C4	125.3 (3)	F1—C23—C24	118.7 (3)
O3—C5—C6	112.9 (2)	F1—C23—C22	118.5 (3)
C4—C5—C6	121.8 (2)	C24—C23—C22	122.8 (2)
C7—C6—C5	120.0 (2)	C23—C24—C25	118.1 (2)
C7—C6—H6A	120.0	C23—C24—H24A	121.0
C5—C6—H6A	120.0	C25—C24—H24A	121.0
C6—C7—C8	120.8 (3)	C24—C25—C20	121.5 (2)
C6—C7—H7A	119.6	C24—C25—H25A	119.2
C8—C7—H7A	119.6	C20—C25—H25A	119.2
C3—C8—C7	119.6 (2)	C31—C26—C27	118.27 (18)
C3—C8—C9	118.9 (2)	C31—C26—C17	119.97 (17)
C7—C8—C9	121.4 (2)	C27—C26—C17	121.75 (19)
C10—C9—C8	121.6 (2)	C28—C27—C26	120.9 (2)
C10—C9—H9A	119.2	C28—C27—H27A	119.5
C8—C9—H9A	119.2	C26—C27—H27A	119.5
C9—C10—C1	118.5 (2)	C29—C28—C27	118.2 (2)
C9—C10—C11	118.9 (2)	C29—C28—H28A	120.9
C1—C10—C11	122.5 (2)	C27—C28—H28A	120.9
C12—C11—C10	126.5 (2)	C28—C29—C30	123.2 (2)
C12—C11—H11A	116.7	C28—C29—F2	118.8 (2)
C10—C11—H11A	116.7	C30—C29—F2	118.0 (2)
C11—C12—C13	122.8 (2)	C29—C30—C31	118.0 (2)
C11—C12—H12A	118.6	C29—C30—H30A	121.0
C13—C12—H12A	118.6	C31—C30—H30A	121.0
O2—C13—C12	122.69 (19)	C30—C31—C26	121.3 (2)
O2—C13—C14	120.62 (19)	C30—C31—H31A	119.3
C12—C13—C14	116.68 (19)	C26—C31—H31A	119.3
C15—C14—C19	119.10 (17)	O3—C32—H32A	109.5
C15—C14—C13	118.22 (17)	O3—C32—H32B	109.5
C19—C14—C13	122.57 (19)	H32A—C32—H32B	109.5
O1—C15—C16	123.75 (19)	O3—C32—H32C	109.5
O1—C15—C14	114.88 (17)	H32A—C32—H32C	109.5
C16—C15—C14	121.29 (18)	H32B—C32—H32C	109.5
C15—C16—C17	119.99 (19)	O1—C33—H33A	109.5
C15—C16—H16A	120.0	O1—C33—H33B	109.5
C17—C16—H16A	120.0	H33A—C33—H33B	109.5
C18—C17—C16	119.15 (17)	O1—C33—H33C	109.5
C18—C17—C26	120.89 (17)	H33A—C33—H33C	109.5
C16—C17—C26	119.90 (18)	H33B—C33—H33C	109.5
C17—C18—C19	121.88 (18)		
			
C10—C1—C2—C3	−0.1 (4)	C14—C15—C16—C17	−1.8 (3)
C1—C2—C3—C8	−2.8 (4)	C15—C16—C17—C18	−0.7 (3)
C1—C2—C3—C4	176.7 (2)	C15—C16—C17—C26	176.75 (18)
C8—C3—C4—C5	0.5 (3)	C16—C17—C18—C19	2.9 (3)
C2—C3—C4—C5	−178.9 (2)	C26—C17—C18—C19	−174.56 (18)
C32—O3—C5—C4	10.3 (4)	C17—C18—C19—C14	−2.4 (3)
C32—O3—C5—C6	−171.2 (3)	C17—C18—C19—C20	177.87 (18)
C3—C4—C5—O3	177.0 (2)	C15—C14—C19—C18	−0.1 (3)
C3—C4—C5—C6	−1.4 (4)	C13—C14—C19—C18	175.87 (18)
O3—C5—C6—C7	−177.7 (2)	C15—C14—C19—C20	179.54 (18)
C4—C5—C6—C7	0.9 (4)	C13—C14—C19—C20	−4.4 (3)
C5—C6—C7—C8	0.4 (4)	C18—C19—C20—C21	133.8 (2)
C2—C3—C8—C7	−179.8 (2)	C14—C19—C20—C21	−45.9 (3)
C4—C3—C8—C7	0.7 (3)	C18—C19—C20—C25	−44.9 (3)
C2—C3—C8—C9	2.7 (3)	C14—C19—C20—C25	135.4 (2)
C4—C3—C8—C9	−176.8 (2)	C25—C20—C21—C22	−1.0 (3)
C6—C7—C8—C3	−1.2 (4)	C19—C20—C21—C22	−179.8 (2)
C6—C7—C8—C9	176.3 (2)	C20—C21—C22—C23	0.1 (4)
C3—C8—C9—C10	0.4 (3)	C21—C22—C23—F1	−178.2 (2)
C7—C8—C9—C10	−177.1 (2)	C21—C22—C23—C24	1.1 (4)
C8—C9—C10—C1	−3.4 (3)	F1—C23—C24—C25	178.1 (2)
C8—C9—C10—C11	172.7 (2)	C22—C23—C24—C25	−1.2 (4)
C2—C1—C10—C9	3.2 (3)	C23—C24—C25—C20	0.1 (3)
C2—C1—C10—C11	−172.7 (2)	C21—C20—C25—C24	1.0 (3)
C9—C10—C11—C12	−167.3 (2)	C19—C20—C25—C24	179.72 (19)
C1—C10—C11—C12	8.5 (4)	C18—C17—C26—C31	131.2 (2)
C10—C11—C12—C13	176.3 (2)	C16—C17—C26—C31	−46.3 (3)
C11—C12—C13—O2	−5.7 (4)	C18—C17—C26—C27	−47.6 (3)
C11—C12—C13—C14	175.1 (2)	C16—C17—C26—C27	135.0 (2)
O2—C13—C14—C15	122.6 (2)	C31—C26—C27—C28	0.2 (3)
C12—C13—C14—C15	−58.2 (3)	C17—C26—C27—C28	179.0 (2)
O2—C13—C14—C19	−53.4 (3)	C26—C27—C28—C29	−0.4 (3)
C12—C13—C14—C19	125.8 (2)	C27—C28—C29—C30	0.0 (3)
C33—O1—C15—C16	−15.3 (3)	C27—C28—C29—F2	179.95 (19)
C33—O1—C15—C14	167.99 (19)	C28—C29—C30—C31	0.5 (3)
C19—C14—C15—O1	179.04 (17)	F2—C29—C30—C31	−179.43 (19)
C13—C14—C15—O1	2.8 (3)	C29—C30—C31—C26	−0.7 (3)
C19—C14—C15—C16	2.3 (3)	C27—C26—C31—C30	0.3 (3)
C13—C14—C15—C16	−173.92 (19)	C17—C26—C31—C30	−178.50 (19)
O1—C15—C16—C17	−178.33 (19)		

### Hydrogen-bond geometry (Å, °)

**Table:**

*Cg*1, *Cg*3 and *Cg*4 are the centroids of the C1–C3/C8–C10, C14–C19 and C20–C25 rings, respectively.

**Table:**

*D*—H···*A*	*D*—H	H···*A*	*D*···*A*	*D*—H···*A*
C28—H28A···O2^i^	0.93	2.53	3.363 (3)	148.
C32—H32C···F1^ii^	0.96	2.40	3.275 (4)	152.
C33—H33A···F2^iii^	0.96	2.48	3.404 (3)	162.
C32—H32A···Cg1^iv^	0.96	2.82	3.767 (4)	168.
C24—H24A···Cg3^v^	0.93	2.83	3.461 (3)	126.
C33—H33B···Cg3^vi^	0.96	2.91	3.556 (3)	126.
C7—H7A···Cg4^iii^	0.93	2.85	3.548 (3)	133.

Symmetry codes: (i) *x*+1, *y*, *z*+1; (ii) *x*+3/2, −*y*+1/2, *z*−1/2; (iii) *x*, *y*, *z*−1; (iv) *x*−1/2, −*y*−1/2, *z*−3/2; (v) *x*−1, *y*, *z*; (vi) −*x*, −*y*, −*z*+1.

## References

[bb1] Astruc, D. (2002). In *Modern Arene Chemistry* Weinheim: Wiley.

[bb2] Baktır, Z., Akkurt, M., Samshuddin, S., Narayana, B. & Yathirajan, H. S. (2011a). *Acta Cryst.* E**67**, o1262–o1263. 10.1107/S1600536811015455 PMC3089231 21754550

[bb3] Baktır, Z., Akkurt, M., Samshuddin, S., Narayana, B. & Yathirajan, H. S. (2011b). *Acta Cryst.* E**67**, o1292–o1293. 10.1107/S160053681101587X PMC3120319 21754699

[bb4] Bruker (2009). *APEX2*, *SAINT* and *SADABS* Bruker AXS Inc., Madison, Wisconsin, USA.

[bb5] Dhar, D. N. (1981). In *The Chemistry of Chalcones and Related Compounds* New York: Wiley.

[bb6] Dimmock, J. R., Elias, D. W., Beazely, M. A. & Kandepu, N. M. (1999). *Curr. Med. Chem.* **6**, 1125–1149. 10519918

[bb7] Fun, H.-K., Hemamalini, M., Samshuddin, S., Narayana, B. & Yathirajan, H. S. (2010*a*). *Acta Cryst.* E**66**, o582–o583. 10.1107/S1600536810004435 PMC2983722 21580348

[bb8] Fun, H.-K., Hemamalini, M., Samshuddin, S., Narayana, B. & Yathirajan, H. S. (2010*b*). *Acta Cryst.* E**66**, o864–o865. 10.1107/S1600536810009414 PMC2983895 21580687

[bb9] Jasinski, J. P., Guild, C. J., Samshuddin, S., Narayana, B. & Yathirajan, H. S. (2010*a*). *Acta Cryst.* E**66**, o1948–o1949. 10.1107/S1600536810026036 PMC3007318 21588274

[bb10] Jasinski, J. P., Guild, C. J., Samshuddin, S., Narayana, B. & Yathirajan, H. S. (2010*b*). *Acta Cryst.* E**66**, o2018. 10.1107/S1600536810026905 PMC3007578 21588329

[bb11] Liu, J. K. (2006). *Chem. Rev.* **106**, 2209–2223. 10.1021/cr050248c 16771447

[bb12] Samshuddin, S., Butcher, R. J., Akkurt, M., Narayana, B., Yathirajan, H. S. & Sarojini, B. K. (2011). *Acta Cryst.* E**67**, o1954–o1955. 10.1107/S1600536811026547 PMC3212338 22090995

[bb13] Samshuddin, S., Narayana, B., Shetty, D. N. & Raghavendra, R. (2011). *Der. Pharm. Chem.*, **3**, 232–240.

[bb14] Sarojini, B. K., Narayana, B., Ashalatha, B. V., Indira, J. & Lobo, K. G. (2006). *J. Cryst. Growth*, **295**, 54–59.

[bb15] Satyanarayana, M., Tiwari, P., Tripathi, B. K., Sriwastava, A. K. & Pratap, R. (2004). *Bioorg. Med. Chem.* **12**, 883–887. 10.1016/j.bmc.2003.12.026 14980600

[bb16] Sheldrick, G. M. (2008). *Acta Cryst.* A**64**, 112–122. 10.1107/S0108767307043930 18156677

[bb17] Spek, A. L. (2009). *Acta Cryst.* D**65**, 148–155. 10.1107/S090744490804362X PMC2631630 19171970

